# Synergistic Regulation of Composition and Growth Kinetics in Cobalt-Doped Nickel Sulfides for High-Performance Pseudocapacitors

**DOI:** 10.3390/ma19122651

**Published:** 2026-06-19

**Authors:** Hung Nguyen Dinh, Cu Dang Van, Thu Thuy Luong Thi, Khu Le Van

**Affiliations:** 1 Faculty of Chemistry, Hanoi National University of Education, Hanoi City 100000, Vietnam; hunghoacvp@vinhphuc.edu.vn (H.N.D.); thuyltt@hnue.edu.vn (T.T.L.T.); 2 Vietnam-Korea Institute of Science and Technology, Hanoi City 100000, Vietnam; dvcu@mst.gov.vn

**Keywords:** Co-doped NiS_2_, sulfur vacancy, flexible carbon cloth, supercapacitor

## Abstract

Transition-metal sulfides are promising electrode materials for high-performance supercapacitors but are often limited by poor conductivity, particle agglomeration, and insufficient active sites. Herein, Co-doped NiS_2_ with tunable sulfur vacancies was directly grown on flexible carbon cloth via a facile one-step solvothermal method by systematically controlling sulfur source ratio, Ni:Co ratio, temperature, and reaction time. Structural analyses reveal that the optimized conditions of S:(Ni + Co) = 3:1, Ni:Co = 2:1, 160 °C, and 15 h promote the formation of phase-pure Co-doped NiS_2_ hierarchical microspheres with enhanced crystallinity and abundant active sites from the synergistic interaction between Ni and Co. Consequently, the optimized electrode delivers an impressive capacitance of 1296 F g^−1^ at a current density of 1 A g^−1^, along with excellent rate performance, retaining more than 88% of its capacitance after 1500 charge/discharge cycles at current densities ranging from 2 to 20 A g^−1^. This work highlights the critical role of synthesis parameter engineering in regulating defect chemistry, structure, and electrochemical performance in advanced energy storage applications.

## 1. Introduction

Given concerns about the depletion of fossil fuel reserves and their associated environmental consequences, the development of renewable energy is inevitable [1]. Due to the intermittent nature and dependence on weather and location of renewable energy sources, energy storage devices are necessary for ensuring a continuous and efficient power supply [2]. In parallel, the rapid growth of portable and wearable electronics has created increasing demand for lightweight, high-performance, and mechanically adaptable energy storage devices [3,4]. Among various energy storage technologies, supercapacitors have attracted considerable attention owing to their high power density, fast charge/discharge capability, and long cycling life [5]. The electrochemical performance of supercapacitors is strongly influenced by the electrode architecture and current collector. Conventional substrates such as nickel foam and stainless steel provide excellent electrical conductivity but are relatively heavy and mechanically weak. In contrast, carbon cloth offers a unique combination of high electrical conductivity, porous structure, low weight, and intrinsic mechanical flexibility, making it an attractive substrate for an advanced supercapacitor electrode [6,7].

On the other hand, supercapacitors generally store charge through two distinct mechanisms: electrochemical double-layer capacitance (EDLC) and pseudocapacitance [8]. In EDLCs, energy storage occurs via electrostatic accumulation of ions at the electrode/electrolyte interface, without charge transfer across the interface, resulting in excellent cycling stability but relatively low capacitance and energy density [9]. In contrast, pseudocapacitors store charge through fast and reversible Faradaic redox reactions occurring at or near the electrode surface [10]. Because these reactions involve electron transfer and utilize a larger fraction of the electrochemically active material, pseudocapacitors can deliver significantly higher specific capacitance and energy density than EDLCs [11,12]. Among electrode materials for pseudocapacitors, transition metal sulfides (TMS), particularly nickel–cobalt sulfides (Ni-Co-S), are of particular interest due to their multiple oxidation states, high theoretical capacitance, and superior conductivity compared to oxides and hydroxides [13]. Chen et al. [14] reported a higher capacitive performance of NiCo_2_S_4_ compared to NiCo_2_O_4_, which is a result of its lower optical band gap energy and higher conductivity. Qi et al. [15] proved that not only the specific capacitance but also the capacitance retention of NiCo_2_S_4_ is higher than that of Ni-Co-LDH (1974.55 mF/cm^2^ and 86.2% of NiCo_2_S_4_ and only 630.77 mF/cm^2^ and 65.3% of Ni-Co-LDH). Even so, Ni-Co-S electrodes face challenges, such as limited conductivity in certain structures and nanomaterial aggregation, both of which reduce the number of active sites. This can be mitigated by controlling the material synthesis conditions, particularly when the appropriate amount of sulfur vacancy is present [16]. Liu et al. [17] investigated the effect of the vacancies on the electrochemical properties, showing that the r-CoNi_2_S_4_ with sulfur vacancies has a capacity 157% higher than the original CoNi_2_S_4_, which is 1918.9 F g^−1^ at a current density of 1 A g^−1^, due to the ability to generate more active sites, shortening the transmission distance of ions and electrons. Lv et al. [18] show that by adjusting the reduction time in the exchange reaction of sulfur and nickel ions, the electrochemical activity of the nickel–cobalt sulfide electrode significantly improves. The CoNi_2_S_4_ electrode exhibits a capacitance of 5.24 F cm^2^ at a current density of 3 mA cm^2^. Jia et al. [19] proved that 6 h of solvothermal treatment at 160 °C and reduction can acquire a hollow structure with better sulfur vacancies, which enabled the electrode to have a higher specific capacity of 763.5 C g^−1^ at 1 A g^−1^ and maintain 91.40% of its capacity after 5000 cycles under 10 A g^−1^. Nevertheless, in these studies, sulfur vacancies are often introduced via several steps, either by forming the hydroxide/oxide and then sulfonating it [16,20] or by reducing it after the synthesis of the Ni-Co-S materials [19,21]. This is time-consuming and chemical-intensive, and it may reduce the amount of Ni-Co-S deposited onto the substrate, especially when synthesizing on flexible substrates.

Addressing these considerations, we herein report the controlled synthesis of Co-doped NiS_2_ directly grown on flexible carbon cloth under tunable solvothermal conditions, thereby establishing a clear mechanistic link between sulfur stoichiometry, Ni-Co synergy, and structural evolution in governing electrochemical performance. The optimal electrode (S:(Ni + Co) = 3:1, Ni:Co = 2:1, 160 °C, and 15 h) exhibits a high specific capacitance of 1296 F g^−1^ at 1 A g^−1^ and more than 88% capacitance retention at current density in the range of 2 and 20 A g^−1^ after 1500 cycles. These results demonstrate that precise control of synthesis parameters can effectively regulate the electrode’s electrochemical properties. Furthermore, the integration of Co-doped NiS_2_ on flexible carbon cloth provides a promising platform for future flexible supercapacitors.

## 2. Materials and Methods

### 2.1. Preparation of Nickel–Cobalt Sulfide on Carbon Cloth

All the reagents and solvents are analytical grade and are used without further purification. Prior to the synthesis, a piece of carbon cloth (CC) (WOS1009, China) (1.0 cm × 3.0 cm) with a thickness of 0.2 mm was carefully cleaned using deionized water, acetone, and absolute ethanol, then wrapped with Teflon tape (Hanoi, Vietnam) to set an active area of 1 cm^2^. The nickel–cobalt sulfide electrode was prepared via a one-step solvothermal reaction. Briefly, solutions of 0.2 M Ni(NO_3_)_2_, 0.2 M Co(NO_3_)_2_, and 0.4 M thiourea in ethanol were mixed with the appropriate ratio (nickel–cobalt = 1:2 ÷ 3:1; thiourea:(nickel + cobalt) = 2:1 ÷ 4:1), then stirred thoroughly for 20 min and then transferred to a Teflon-lined autoclave. A piece of pre-cleaned CC was then immersed in the mixture as the substrate. The solvothermal temperatures were set at 140, 150, 160, and 170 °C, with specified durations of 9, 12, 15, and 18 h. After cooling, it was washed with hot double-distilled water and ethanol, then dried at 80 °C overnight. The resulting samples were labeled as N_x_C_y_S_z_-a-b, where “x”, “y”, and “z” represent the precursor molar ratio of Ni, Co, and thiourea; “a” corresponds to the solvothermal temperature in °C; and “b” denotes the reaction time in hours.

### 2.2. Characterization

The morphologies, microstructures, and elemental composition of the synthesized NiCoS over CC were analyzed using field-emission scanning electron microscopy (S-4800, HITACHI, Japan ) equipped with EDS (7593-H, HORIBA, Northampton, UK), X-ray diffractometer (D8 Advance, BRUKER, Germany, Cu Kα radiation, λ = 1.5406 Å), and Raman spectra (LabRAM HR Evolution, HORIBA, France, 532 nm excitation laser).

### 2.3. Electrochemical Measurements

Cyclic voltammetry (CV), galvanostatic charge–discharge (GCD) test, and electrochemical impedance spectroscopy (EIS) were performed on a Potentiostat/Galvanostat (VSP, BioLogic, France) in a 3.0 KOH aqueous solution at room temperature. The measurements were conducted with a three-electrode configuration, in which nickel–cobalt sulfide on CC, a Pt foil, and a 1 M KOH HgO/Hg electrode served as the working, counter, and reference electrodes, respectively. The capacitance was determined from CV curves and GCD curves using Equations (1) and (2), respectively:

(1) $$C_{CV}=\int \frac{IdV}{mv\Delta V}$$

(2) $$C_{GCD}=\frac{\left|I\right|\Delta t}{2m\Delta V}$$

where *I* is the current intensity, *v* is the scan rate, Δ*t* is the time corresponding to each charge–discharge cycle, Δ*V* is the potential window, and *m* is the mass of the active material in the electrode.

## 3. Results

### 3.1. Morphological and Structural Characterization

#### 3.1.1. Morphology

**Figure 1:**
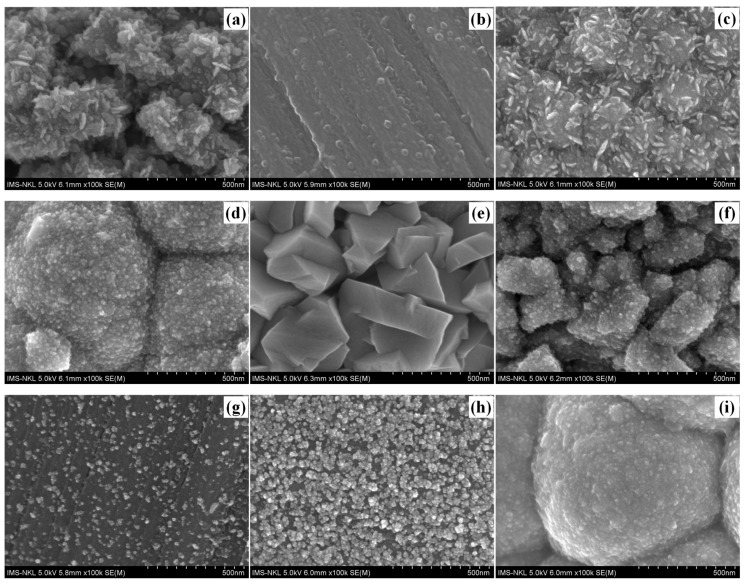
SEM images of nickel–cobalt sulfide (**a**) N_2_C_1_S_6_-160-15, (**b**) N_2_C_1_S_12_-160-15, (**c**) N_1_C_2_S_9_-160-15, (**d**) N_2.25_C_0.75_S_9_-160-15, (**e**) N_2_C_1_S_9_-140-15, (**f**) N_2_C_1_S_9_-170-15, (**g**) N_2_C_1_S_9_-160-9, (**h**) N_2_C_1_S_9_-160-18, (**i**) N_2_C_1_S_9_-160-15.

The SEM results collectively demonstrate that the S:(Ni + Co) ratio, the Ni:Co composition ratio, synthesis temperature, and reaction time cooperatively regulate the nucleation-growth kinetics, thereby dictating the final morphology of the obtained materials. As summarized in Figure 1, these parameters sequentially influence nucleation density, growth pathway, and structural evolution, ultimately leading to an optimized hierarchical architecture, while intermediate states are provided in the Supporting Information (Figure S1).

The S:(Ni + Co) ratio plays a decisive role in determining nucleation behavior at the early stage of synthesis. At a relatively low S:(Ni + Co) ratio of 2 (Figure 1a), the surface is dominated by a limited number of nucleation sites, manifested as sparsely distributed nanoparticles. In contrast, increasing the thiourea:(Ni + Co) ratio to 4 (Figure 1b) significantly increases nucleation density, resulting in densely packed nanoparticle aggregates. The surface becomes highly rough and composed of interconnected nanograins, indicating rapid nucleation followed by particle growth and coalescence. Following nucleation, the Ni:Co ratios govern the crystal growth pathway. As shown in Figure 1c,d, a Co-rich composition (Ni:Co = 1:2) favors anisotropic growth, forming loosely assembled platelet-like structures, whereas a Ni-rich composition (Ni:Co = 3:1) results in dense and compact morphologies due to enhanced isotropic growth and particle fusion. At intermediate ratios (Ni:Co = 1:1, Figure S1a), a gradual transition toward well-defined microspherical architectures is observed.

The synthesis temperature further modulates the growth kinetics and structural refinement. At 140 °C (Figure 1e), the morphology is dominated by incomplete and irregular structures, reflecting kinetically limited growth. Increasing the temperature leads to progressive structural evolution (Figure S1b), in which nanoparticle aggregation and partial spherical assembly begin to merge. At 170 °C (Figure 1f), accelerated crystallite growth and interparticle fusion result in dense structures with reduced surface roughness. Reaction time dictates the temporal evolution from nucleation to structural stabilization. At an early stage (9 h, Figure 1g), only isolated nanoparticles are observed, indicating incomplete nucleation. With increasing reaction time (12 h, Figure S1c), particle density and connectivity improve, accompanied by gradual self-assembly into a microspherical structure. In contrast, the long reaction time of 18 h (Figure 1h) leads to structural coarsening and partial collapse of the hierarchical architecture, likely due to Ostwald ripening and overgrowth.

Overall, the results establish a clear structure-evolution relationship, in which the S:(Ni + Co) ratio controls nucleation density, the Ni:Co ratio directs growth pathways, temperature regulates kinetic processes, and reaction time governs structural evolution. The optimized condition (S:(Ni + Co) = 3:1, Ni:Co = 2:1, 160 °C, and 15 h) represents a critical balance among these factors, enabling controlled nucleation, isotropic growth, and stable self-assembly into highly uniform and well-defined microspheres with hierarchical surfaces constructed from fine nanoparticles (Figure 1i). Such architectures, featuring high surface roughness and structural coherence, are expected to provide enhanced accessibility of active sites and improved performance in electrochemical applications.

#### 3.1.2. Elemental Composition

**Figure 2:**
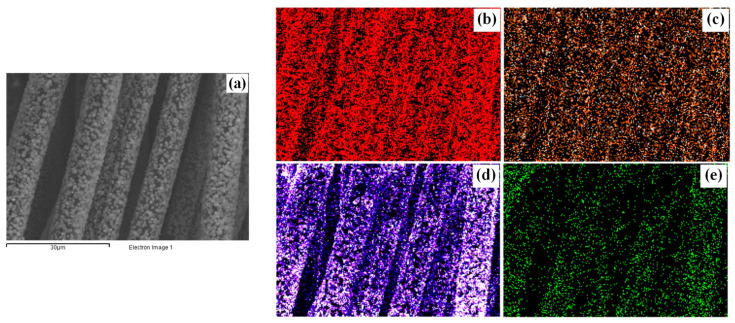
(**a**) Electron image and EDS layer images: (**b**) of Ni, (**c**) of Co, (**d**) of S, and (**e**) of O for N_2_C_1_S_9_-160-15.

Energy-dispersive X-ray spectroscopy (EDX) was employed to examine the elemental composition and stoichiometry of the synthesized materials. The representative spectrum and element mapping of the optimized sample (N_2_C_1_S_9_-160-15) in Figure 2 confirm the presence of Ni, Co, and S as the dominant elements, with uniform spatial distribution. Quantitative analysis in Table 1 reveals that the sulfur-to-metal ratio (S:(Ni + Co)) ranges from 1.14 to 1.80, depending on the synthesis conditions, and is generally lower than the ideal value of 2 for NiS_2_. For samples with varying Ni:Co ratios (from N_1_C_2_S_9_-160-15 to N_2_C_1_S_9_-160-15), sulfur content remains relatively consistent at 1.5–1.6, suggesting the formation of a slightly sulfur-deficient sulfide phase. At higher Ni content (N_3_C_1_S_9_-160-15), the sulfur ratio further decreases (S:(Ni + Co) = 1.38), indicating an increase in compositional imbalance. More importantly, the actual Ni fraction (Ni/(Ni + Co) increases from 0.51 in N_1_C_2_S_9_-160-15 to 0.67, 0.82 and 0.89 for N_1.5_C_1.5_S_9_-160-15, N_2_C_1_S_9_-160-15 and N_2.25_C_0.75_S_9_-160-15, respectively. This indicates that Ni is preferentially incorporated into the sulfide phase, while Co incorporation becomes progressively limited as the Ni content increases. Such behavior suggests differences in sulfuration kinetics and metal-sulfur affinity, with Ni species forming more stable sulfide frameworks under the given solvothermal conditions. On the other hand, the S:(Ni + Co) ratio plays a critical role in determining stoichiometry. Reducing the thiourea ratio (N_2_C_1_S_6_-160-15) leads to significant sulfur deficiency (S:(Ni + Co) = 1.14), whereas increasing it (N_2_C_1_S_12_-160-15) enhances sulfur incorporation (S:(Ni + Co) = 1.77), approaching the stoichiometric limit. This indicates that sulfur availability governs the extent of sulfide formation.

A similar dependence is observed for solvothermal temperature. At a lower temperature (140 °C), the sulfur content is relatively low (S:(Ni + Co) = 1.22), suggesting incomplete reaction. Increasing the temperature to 150–160 °C improves sulfur incorporation (S:(Ni + Co) = 1.49–1.53), whereas further increasing it to 170 °C results in a decrease (1.20), suggesting possible sulfur loss at elevated temperatures. The reaction time also significantly affects the composition. A short reaction time (9–12 h) results in a high oxygen content, indicating incomplete conversion and surface oxidation, whereas the sample synthesized at 15 h shows lower oxygen content and a more balanced composition (S:(Ni + Co) = 1.53). A prolonged reaction (18 h) results in an increase in oxygen content and a slight deviation in the sulfur ratio, suggesting partial oxidation or structural degradation.

#### 3.1.3. Crystalline Structure

**Figure 3:**
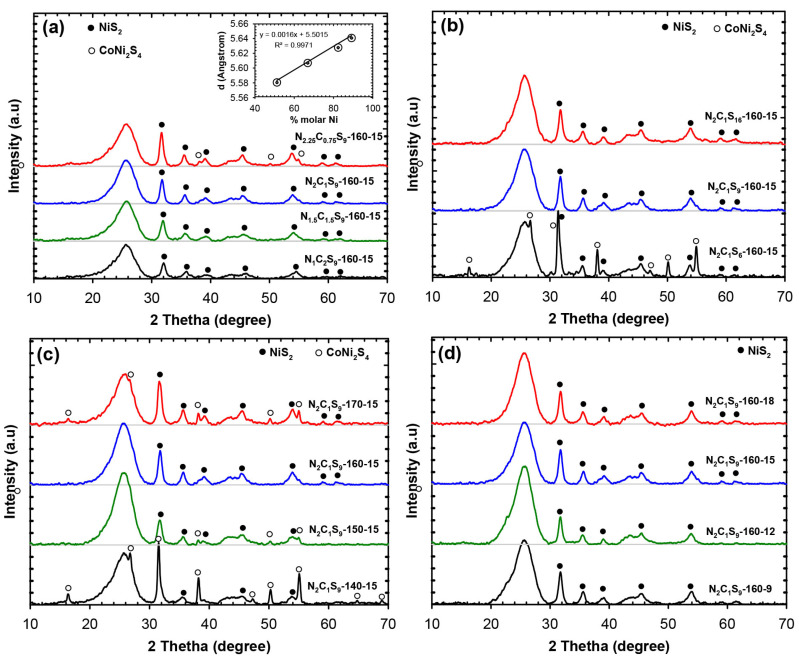
XRD patterns of nickel–cobalt sulfide growth on carbon cloth synthesized under different solvothermal conditions: (**a**) Ni:Co ratio, (**b**) S:(Ni + Co) ratio, (**c**) temperature, and (**d**) time.

To elucidate the phase evolution and determine the effect of synthesis conditions on the crystal structure of all samples prepared, XRD was systematically investigated. As shown in Figure 3, all samples exhibit a series of characteristic reflections located at approximately 31.76°, 35.39°, 39.10°, 45.42°, and 53.92°, which can be indexed to the (200), (210), (211), (220), and (311) planes of cubic NiS_2_ [22], confirming that NiS_2_ is the dominant crystalline phase in the obtained materials. In addition, a broad diffraction peak centered near 26° is observed for all samples, corresponding to the (002) plane of graphitic carbon originating from the carbon cloth substrate [23]. The overall diffration patterns indicate that the pyrite-type NiS_2_ framework is preserved across the entire composition range.

The influence of metal precursor composition was first examined by varying the Ni:Co ratio (1:2, 1:1, 2:1, and 3:1) under identical solvothermal conditions. Although the principal NiS_2_ reflections remain unchanged (Figure 3a), noticeable variations in peak intensity and width are observed. Generally, as the Ni:Co ratio increases, the diffraction peaks gradually become sharper and more intense. Scherrer analysis of the (002) reflection yielded crystallite sizes of 9.167, 8.559, 10.383, and 10.468 nm for N_1_C_2_S_9_-160-15, N_1.5_C_1.5_S_9_-160-15, N_2_C_1_S_9_-160-15, and N_2.25_C_0.75_S_9_-160-15, respectively, further indicating improved crystallinity and more complete development of the sulfide lattice at higher Ni concentrations.

As the Ni:Co ratio increases from 1:2 to 2:1, the diffraction peaks gradually become sharper and more intense, indicating improved crystallinity and more complete development of the sulfide lattice. Further increasing the Ni content to Ni:Co = 3:1 results in slight peak broadening (Figure 3a), suggesting the introduction of lattice disorder or a reduced crystallite size due to excessive Ni incorporation.

While S:(Ni + Co) ratio and solvothermal temperature play critical roles in determining the phase purity of the Ni-Co sulfide system. As shown in Figure 3b,c, the N_2_C_1_S_6_-160-15 and N_2_C_1_S_9_-140-15 exhibit several weak additional reflections at approximately 16.38°, 26.74°, 38.18°, and 50.26°, which can be indexed to the (111), (220), (400), and (551) planes of spinel CoNi_2_S_4_ (JCPDS No. 24-0334). The appearance of these reflections indicates that insufficient thermal energy or sulfur-deficient conditions lead to incomplete phase transformation, resulting in the coexistence of spinel domains with the NiS_2_ phase. Increasing the reaction temperature to 150 °C significantly suppresses these secondary reflections while enhancing the intensity of the NiS_2_ diffraction peaks, suggesting improved crystallization and higher phase purity (Figure 3c). At 160 °C, the characteristic NiS_2_ peaks become the sharpest and most intense, while the reflections associated with CoNi_2_S_4_ are no longer detectable, indicating the formation of phase-pure Co-doped NiS_2_. However, when the temperature is further increased to 170 °C, several weak additional reflections reappear, accompanied by slight peak broadening, suggesting that excessive thermal energy may induce partial phase segregation or structural disorder. A similar crystallization trend is observed as the solvothermal reaction time is varied: peak intensity increases from 9 to 15 h and slightly broadens at 18 h, suggesting that prolonged reaction time may introduce minor lattice distortion (Figure 3d). Based on these results, the optimal synthesis conditions for the formation of phase-pure Co-doped NiS_2_ are determined to be Ni:Co = 2:1, thiourea:(Ni:Co) = 3:1.

A closer examination of the diffraction patterns reveals a gradual shift of the (200) reflections toward lower diffraction angles as the Ni fraction increases. Using the (200) peak position, the calculated lattice parameters increase from approximately 5.5804 ± 0.0010 to 5.6407 ± 0.0007 across the sample series from N_1_C_2_S_9_-160-15 to N_2.25_C_0.75_S_9_-160-15 (detail in Table S1), indicating systematic lattice expansion. Such a monotonic variation is consistent with the behavior predicted by Vegard’s law and suggests the formation of a substitutional solid solution within the pyrite-type NiS_2_ lattice (insert of Figure 3a). The observed lattice expansion can be rationalized by the partial replacement of smaller Co ions by larger Ni ions at the metal sites of the pyrite framework [14], which slightly increases the metal-sulfur bond distances and results in expansion of the cubic unit cell.

#### 3.1.4. Chemical Bonding Characteristics

**Figure 4:**
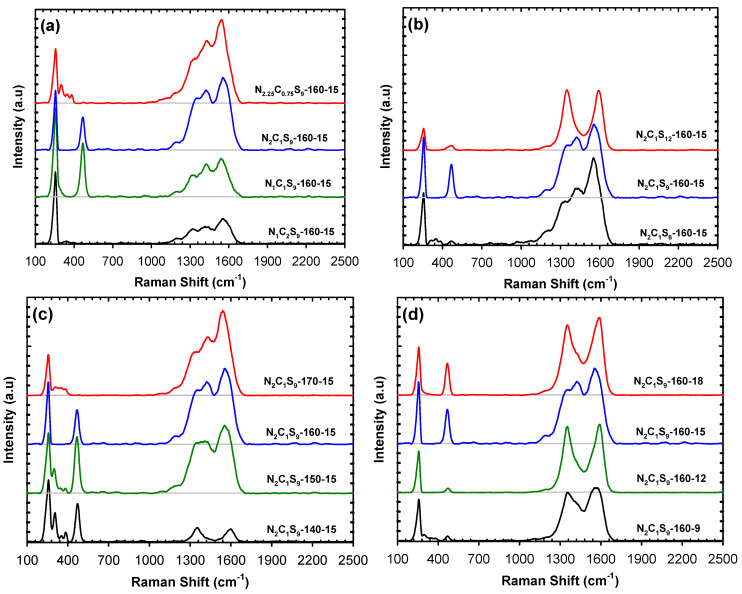
Raman spectra of nickel–cobalt sulfide growth on carbon cloth synthesized under different solvothermal conditions: (**a**) Ni:Co ratio, (**b**) S:(Ni + Co) ratio, (**c**) temperature, and (**d**) time.

To further probe the local bonding environment and defect structure beyond the long-range crystallinity revealed via XRD, Raman spectroscopy was performed. As shown in Figure 4, besides the characteristic D and G bands of carbon cloth located at approximately 1350 and 158 cm^−1^, all samples exhibit three characteristic Raman bands located at approximately 280–285, 380–390, and 470–480 cm^−1^, which can be assigned to the E_g_, A_g_, and T_g_ vibrational modes of the pyrite-type NiS_2_ structures [24,25], respectively. The E_g_ mode originates from the librational motion of S_2_^2-^ dumbbells, while the A_g_ and T_g_ modes correspond to symmetric and antisymmetric stretching of S−S bonds within the disulfide units [26,27]. More importantly, the evolution of Raman features provides insight into the defect chemistry and structural disorder induced by different synthesis parameters. With varying Ni:Co ratios, a subtle red shift of the A_g_ and T_g_ modes is observed as the Ni content increases, which can be attributed to lattice expansion and the modulation of metal-sulfur bonding strength resulting from the substitution of Co by Ni (Figure 4a). This observation is consistent with the lattice parameter expansion derived from XRD analysis. However, in the Ni-rich sample (N_2.25_C_0.75_S_9_-160-15), T_g_ mode nearly disappears, indicating increased disorder and compositional deviation, consistent with the EDX results.

Under sulfur-deficient conditions (N_2_C_1_S_6_-160-15), the Raman peaks, particularly the A_g_ mode at 385 cm^−1^, become broadened, indicating a disruption of the S-S bonding environment due to the formation of sulfur vacancies. (Figure 4b) These defects weaken the lattice’s vibrational coherence and introduce local disorder. In contrast, the sulfur-rich sample (N_2_C_1_S_12_-160-15) exhibits slight blue shifts and attenuated Raman intensity, which can be attributed to lattice distortion or the formation of non-stoichiometric sulfide domains. Samples synthesized at lower temperature (N_2_C_1_S_9_-140-15) (Figure 4c) or shorter reaction time (N_2_C_1_S_9_-160-9 and N_2_C_1_S_9_-160-12) (Figure 4d) display weak and broadened Raman features, reflecting incomplete crystallization and poorly developed S_2_ units. Increasing the temperature to 150–160 °C and extending the reaction time to 15 h significantly enhances peak intensity and reduces peak width, indicating improved crystallinity and lattice ordering. However, a further increase to 170 °C or a prolonged reaction time (18 h) results in peak broadening and a loss of intensity, suggesting the onset of sulfur loss, defect accumulation, or structural degradation.

### 3.2. Electrochemical Performance

**Figure 5:**
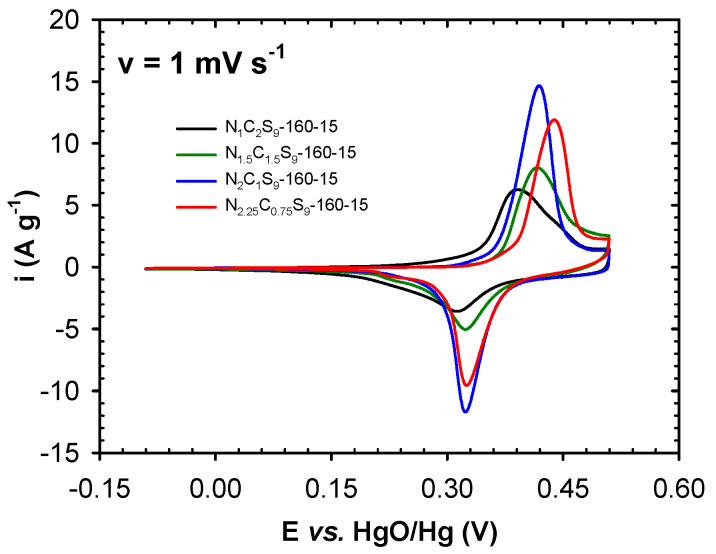
CV curves at 1 mV s^−1^ of nickel–cobalt sulfide electrodes synthesized under different Ni:Co ratios.

The electrochemical performance of the Co-NiS2 electrode with varying Ni/Co ratios was first investigated via cyclic voltammetry. The obtained CV curves are shown in Figure 5 and Figure S2. It can be seen that a pair of pronounced redox peaks within 0.25–0.45 V (vs. HgO/Hg), characteristic of Faradaic reactions involving Ni^2+^/Ni^3+^ and Co^2+^/Co^3+^ [28,29]. Notably, both the peak position and intensity are strongly modulated by the Ni-Co interaction. As the Ni content increases from N_1_C_2_S_9_-160-15 to N_2_C_1_S_9_-160-15, the anodic and cathodic peaks become significantly sharper and more intense, accompanied by slight shifts toward lower overpotential, indicating reduced polarization and improved reaction reversibility. This evolution correlates well with the calculated electrochemically active site density, which increases from 3.731 to 3.767 μmmol g^−1^ for N_1_C_2_S_9_-160-15 and N_1.5_C_1.5_S_9_-160-15, respectively, and reaches a maximum of 8.045 μmol g^−1^ for N_2_C_1_S_9_-160-15 (Figure S3), confirming that the synergistic coupling between Ni and Co maximizes the number of accessible redox centers. The N_2_C_1_S_9_-160-15 electrode therefore exhibits the highest peak current density and the most symmetric redox features, reflecting optimal charge-transfer kinetics and redox utilization. In contrast, the Co-rich sample (N_1_C_2_S_9_-160-15) shows broader peaks at shifted potentials, with lower current responses, consistent with its limited active-site density and sluggish kinetics. Upon further increasing the Ni content to N_2.25_C_0.75_S_9_-160-15, the peak intensity decreases, accompanied by a reduction in the active site to 4.614 μmol g^−1^, indicating that excessive Ni disrupts optimal electronic interactions and reduces redox accessibility.

**Figure 6:**
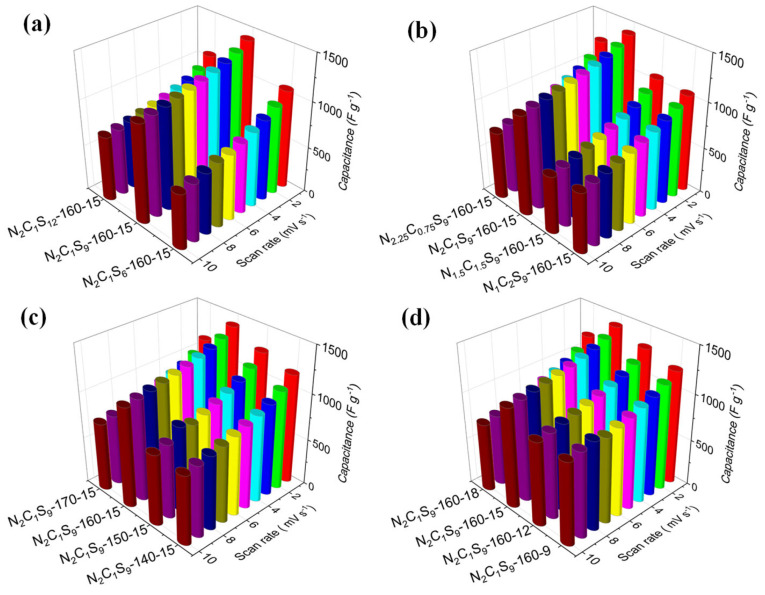
Capacitance calculated from the CV curves of the nickel–cobalt sulfide electrodes synthesized under different solvothermal conditions: (**a**) S:(Ni + Co) ratio, (**b**) Ni:Co ratio, (**c**) temperature, and (**d**) time.

The capacitances of all electrodes, calculated from the CV curves (Figure S2) and the GCD curves (Figure S4), are shown in Figure 6 and Table 2. The effect of S:(Ni + Co) ratio on capacitive performance was first investigated. As sulfur content increases from deficient to excess conditions, capacitance shows a pronounced nonlinear evolution (Figure 6a and Table 2). The sulfur-deficient sample (N_2_C_1_S_6_-160-15) delivers 1072 F g^−1^ at 1 mV s^−1^, which gradually decreases to 962, 885, 823, and 769 F g^−1^ as the scan rate increases from 2 to 5 mV s^−1^, and further drops to 605 F g^−1^ at 10 mV s^−1^, indicating sluggish ion diffusion and limited utilization of active sites. Further increasing to sulfur-rich conditions (N_2_C_1_S_4_-160-15) results in reduced performance (1077, 968, 905, 860, 824, and 795 F g^−1^ at 1–6 mV s^−1^, declining to 709 F g^−1^ at 10 mV s^−1^). GCD measurements further confirm this trend, where capacitance values evolve from 949, 856, 805, 771, and 723 F g^−1^ (sulfur-deficient) to 996, 919, 857, 806, and 768 F g^−1^ (sulfur-rich) at a current density of 1–5 A g^−1^. Notably, both sulfur-deficient and sulfur-excess samples show faster capacitance decay as the scan rate and current density increase, indicating inferior rate capability. This behavior is consistent with previous reports in transition metal sulfides, where moderate sulfur deficiency is known to introduce sulfur vacancies that enhance electronic conductivity and create additional redox-active sites, while excessive sulfur deficiency leads to incomplete sulfide formation and poor electrical connectivity, and sulfur-rich conditions induce lattice disorder and impede charge transport (as reported later). The present results clearly confirm that an optimal sulfur stoichiometry is required to balance defect generation and structural stability.

A similarly nonlinear dependence is observed as the Ni:Co ratio is varied while the sulfur ratio is held fixed at 3 (Figure 6b and Table 2). The Co-rich sample (N_1_C_2_S_9_-160-15) exhibits relatively low capacitance, decreasing from 1053 F g^−1^ at 1 mV s^−1^ to 978, 916, 862, and 816 F g^−1^ at 2–5 mV s^−1^, respectively, and further to 658 F g^−1^ at 10 mV s^−1^, indicating limited redox activity and poor rate performance. Increasing the Ni content to an equimolar ratio (N_1.5_C_1.5_S_9_-160-15) results in only a marginal improvement (1079, 983, 897, 835, 628 F g^−1^ at scan rates of 1–10 mV s^−1^, respectively), suggesting that simply adding Ni does not immediately translate into enhanced performance. Further increasing the Ni fraction (N_2.25_C_0.75_S_9_-160-15) leads to a more pronounced enhancement (1218, 1076, 1001, 946, and 902 F g^−1^ at 1–5 mV s^−1^, respectively, with 729 F g^−1^ at 10 mV s^−1^), yet it still falls short of the maximum observed at the intermediate composition. GCD results follow the same trend, increasing from 935–749 F g^−1^ (Co-rich) to 983–753 F g^−1^ (equimolar) and 1080–874 F g^−1^ (Ni-rich), indicating that a balanced Ni-Co ratio is essential to maximize the synergistic effect between Ni redox activity and Co-induced electronic modulation.

The synthesis conditions, including both temperature and reaction time, further modulate electrochemical performance by influencing crystallinity and sulfur incorporation (Figure 6c and Table 2). At 140 °C (N_2_C_1_S_9_-140-15), the capacitance is relatively limited, decreasing from 1183 F g^−1^ at 1 mV s^−1^ to 1070, 996, 942, and 898 F g^−1^ at 2–5 mV s^−1^, respectively, and further to 749 F g^−1^ at 10 mV s^−1^ due to incomplete crystallization and insufficient activation of electrochemical sites. Increasing the temperature to 150 °C (N_2_C_1_S_9_-150-15) improves performance (1264, 1161, 1081, 965, and 769 F g^−1^ at 1–10 mV s^−1^, respectively), indicating enhanced crystallinity and sulfur incorporation. However, further increasing the temperature to 170 °C (N_2_C_1_S_9_-170-15) results in a noticeable decline in the values (1145, 1030, 936, 915, 876, and 739 F g^−1^ at 1–10 mV s^−1^, respectively). GCD measurements exhibit a consistent trend, with capacitance values evolving from 1103–849 F g^−1^ (140 °C) to 1150–922 F g^−1^ (150 °C), then decreasing to 1045–823 F g^−1^ (170 °C). A similar volcano-type dependence is observed with reaction time. At shorter duration (9 h, N_2_C_1_S_9_-160-9), the capacitances remain relatively low (1224 to 891 F g^−1^ at 1 to 10 mV s^−1^, and 1082 to 896 F g^−1^ at 1 to 5 A g^−1^). At 12 h, the N_2_C_1_S_9_-160-12 exhibits improved capacitances of 1314 F g^−1^ at 1 mV s^−1^ and 1195 F g^−1^ at 1 A g^−1^. However, further prolonging the reaction time to 18 h results in a deterioration in performance (1188 F g^−1^ at 1 mV s^−1^ and 1055 F g^−1^ at 1 A g^−1^), confirming volcano-type behavior (Figure 6d and Table 2).

The above volcano-type trends clearly identify the N_2_C_1_S_9_-160-15 samples as the optimal material, exhibiting the highest capacitance and best rate capability among all investigated conditions. This sample delivers an outstanding capacitance of 1413 F g^−1^ at 1 mV s^−1^ and retains 1080 F g^−1^ at 10 mV s^−1^, along with excellent GCD performance of 1296–1139 F g^−1^ at 1–5 A g^−1^. Such superior electrochemical behavior originates from the synergistic optimization of sulfur stoichiometry, Ni-Co composition, and synthesis temperature and reaction time. Specifically, the intermediate sulfur content ensures an optimal sulfur vacancy concentration, enhancing conductivity without compromising structural integrity, while the balanced Ni:Co ratio maximizes the coupling between redox activity and electronic-structure modulation. Meanwhile, the synthesis temperature of 160 °C and reaction time of 15 min enable the formation of a well-crystallized yet nanostructured material with high purity. As a result, the N_2_C_1_S_9_-160-15 sample achieves an ideal balance between composition and structure, resulting in exceptional capacitive performance.

**Figure 7:**
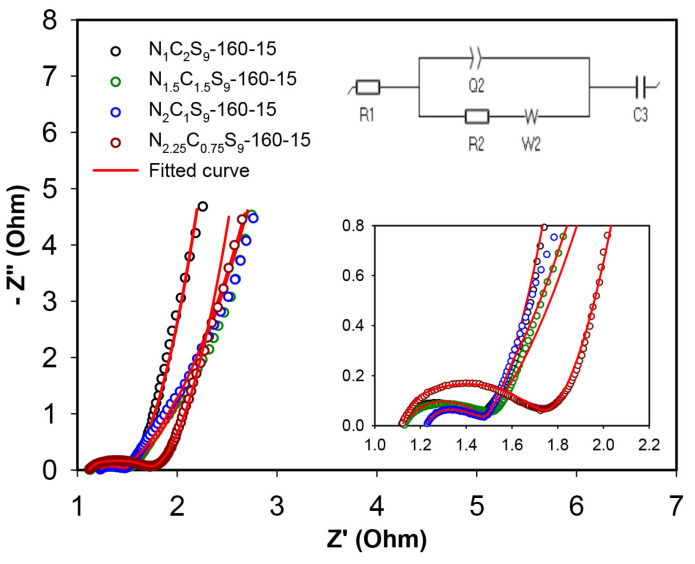
Nyquist plot of nickel–cobalt sulfide electrodes synthesized under different Ni:Co ratios (Inset shows the enlarged view of the Nyquist plot in the high frequency region, and the equivalent circuit model fitted the Nyquist data).

To gain deeper insight into the charge-storage mechanism and ion/electron transport behavior, electrochemical impedance spectroscopy was further analyzed systematically. The Nyquist plots and equivalent circuit model [30,31] fitted to the Nyquist data are shown in Figure 7 and Figure S4. In the equivalent circuit model, R_1_ represents the solution resistance associated with the electrolyte, current collector, and contact interfaces. R_2_ corresponds to the charge-transfer resistance and reflects the kinetics of Faradaic redox reactions. Q_2_ and its exponent a_2_ describe the behavior of the non-ideal double-layer capacitance. The Warburg coefficient (σ_s_) is related to ion diffusion, while C_3_ represents the pseudocapacitive contribution associated with reversible surface redox reactions. The fitted results are summarised in Table 3. Among the samples, the optimized composition (N_2_C_1_S_9_-160-15) consistently delivers the lowest charge-transfer resistance (R_2_ = 0.237 Ω cm^2^), indicating significantly enhanced interfacial charge-transfer kinetics. In contrast, both sulfur-deficient (N_2_C_1_S_6_-160-15) and sulfur-rich samples (N_2_C_1_S_12_-160-15) exhibit substantially higher resistance values (R_2_ = 0.892 and 0.864 Ω cm^2^, respectively). This behavior suggests that sulfur stoichiometry plays a critical role in regulating the electronic structure. Moderate sulfur deficiency introduces an optimal concentration of sulfur vacancies, enhancing electronic conductivity and creating additional active sites. However, excessive sulfur deficiency leads to incomplete sulfide formation and poor electrical connectivity, while sulfur-rich conditions induce lattice disorder and hinder charge transport.

A similar volcano-type trend is observed as the Ni:Co ratio is varied. The charge-transfer resistance decreases markedly from 0.398 Ω cm^2^ in the Co-rich sample (N_1_C_2_S_9_-160-15) to 0.237 Ω cm^2^ at the optimal Ni:Co ratio of 2:1, followed by an increase at higher Ni content (0.779 Ω cm^2^ for N_2.25_C_0.75_S_2_-160-15). This evolution closely matches the variation in capacitance, indicating that the synergistic interaction between Ni and Co governs both charge transfer kinetics and electrochemical activity. The optimized composition achieves a balance among redox activity, electronic conductivity, and structural stability, thereby minimizing interfacial resistance and maximizing electrochemical performance. This is further supported by the relatively high constant phase element exponent (a_2_ = 0.773 for N_2_C_1_S_6_-160-15), indicating a more homogeneous electrode surface and near-ideal capacitive behavior.

The influence of synthesis temperature and reaction time further reinforces this correlation. Both lower (140 °C) and higher (170 °C) temperatures, or shorter (9 h) and longer (18 h) reaction times, lead to increased R_2_ values and reduced electrochemical performance. The higher resistance at low temperature and short reaction time can be attributed to incomplete crystallization and insufficient particle connectivity, while the deterioration at high temperature and prolonged reaction time is associated with particle coarsening, sulfur loss, and particle oxidation, as evidenced by XRD and EDX analysis. These structural and compositional deviations reduce the number of accessible active sites and increase efficiency.

**Figure 8:**
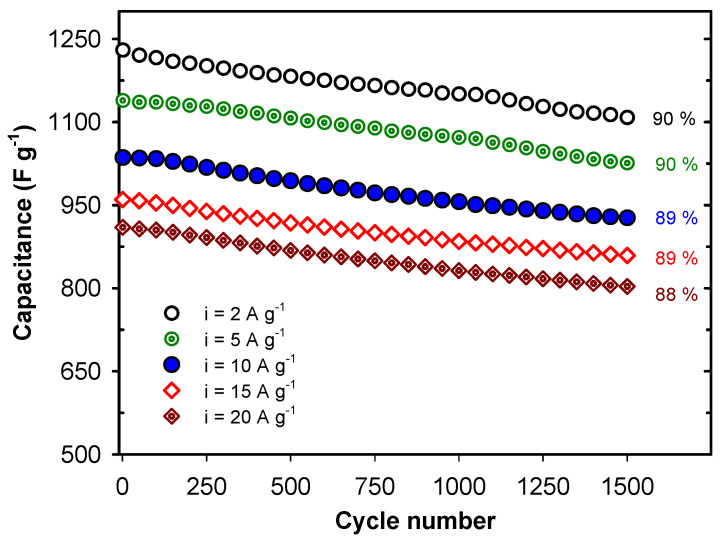
Cycling performance of N_2_C_1_S_9_-160-15 electrode at 2, 5, 10, 15, and 20 A g^−1^.

Figure 8 presents the GCD cycling measurements for the N_2_C_1_S_9_-160-15 sample at current densities ranging from 2 to 20 A g^−1^. The specific capacitances achieved were 1230, 1139, 1036, 960, and 910 F g^−1^ at current densities of 2, 5, 10, 15, and 20 A g^−1^, respectively. Notably, the high specific capacitance of 910 F g^−1^ at a current density of 20 A g^−1^ highlights the material’s excellent rate capability and power density as an electrode. Cycling performance is another key factor for evaluating a supercapacitor. As shown in Figure 8, the material exhibits a good electrochemical performance, maintaining over 88% of its initial capacitance after 1500 cycles. These results demonstrate the significant potential of N_2_C_1_S_9_-160-15 for practical applications.

The comparison of results from this study with those of other similar nickel–cobalt sulfide studies in Table 4 reveals that the as-prepared material exhibits outstanding electrochemical properties, coupled with remarkably straightforward synthesis conditions. It shows competitiveness not only with similar materials synthesized on flexible substrates but also with those produced on rigid substrates.

## 4. Conclusions

In summary, a systematic investigation of Co-doped NiS_2_ synthesized under tunable solvothermal conditions establishes a clear mechanistic link between sulfur stoichiometry, Ni-Co synergy, and structural evolution in governing electrochemical performance. Sulfur precursor ratio controls nucleation density and defect formation, while the Ni:Co ratio dictates the crystal growth pathway and electronic interactions, and reaction temperature and time regulate crystallinity and phase purity. An optimal balance among these parameters (S:(Ni + Co) = 3:1, Ni:Co = 2:1, 160 °C and 15 h) enables the formation of hierarchical microspheres composed of interconnected nanograins with appropriate sulfur vacancy concentration and enhanced electronic conductivity. As a result, the optimized electrode delivers a high capacitance exceeding 1400 F g^−1^ at low scan rate in cyclic voltammetry and maintains 1080 F g^−1^ at 10 mV s^−1^, while galvanostatic charge–discharge measurements yield capacitance values of 1296 F g^−1^ to 1139 F g^−1^ over a current density of 1–5 A g^−1^, demonstrating excellent rate capability. In contrast, both sulfur-deficient and sulfur-excess conditions, as well as imbalanced Ni/Co ratios, lead to reduced capacitance and faster performance decay, highlighting a pronounced volcano-type dependence. Electrochemical impedance analysis further confirms that the optimized composition achieves the lowest charge-transfer resistance, arising from the synergistic coupling between Ni and Co and the maximized density of electrochemically active sites. These findings demonstrate that integrating defect engineering with metal synergy and kinetic control provides a powerful strategy for tailoring structure–property relationships in transition-metal sulfides, offering general design guidelines for developing high-performance electrochemical energy storage materials.

## Figures and Tables

**Table 1:** EDS analysis of the samples.

Samples	% Wt. (%)				Molar Ratio
	Ni	Co	S	O	S:(Ni:Co)
N_1_C_2_S_9_-160-15	26.40	25.14	44.33	4.12	1.58:(0.51:0.49)
N_1.5_C_1.5_S_9_-160-15	34.70	17.20	43.00	5.10	1.52:(0.67:0.33)
N_2_C_1_S_9_-160-15	43.09	9.24	43.78	3.89	1.53:(0.82:0.18)
N_2.25_C_0.75_S_9_-160-15	49.26	5.99	41.57	3.19	1.38:(0.89:0.11)
N_2_C_1_S_6_-160-15	47.52	8.88	35.04	8.55	1.14:(0.84:0.16)
N_2_C_1_S_12_-160-15	38.34	8.08	44.74	8.85	1.77:(0.83:0.17)
N_2_C_1_S_9_-140-15	48.62	11.11	39.67	0.60	1.22:(0.81:0.19)
N_2_C_1_S_9_-150-15	40.27	8.35	39.41	11.97	1.49:(0.83:0.17)
N_2_C_1_S_9_-170-15	47.43	10.96	38.30	3.30	1.20:(0.81:0.19)
N_2_C_1_S_9_-160-9	37.16	4.92	41.38	16.54	1.80:(0.88:0.12)
N_2_C_1_S_9_-160-12	38.25	5.82	38.63	17.30	1.61:(0.87:0.13)
N_2_C_1_S_9_-160-18	33.31	7.59	36.24	22.85	1.62:(0.81:0.19)

**Table 2:** Specific capacitance calculated from GCD curves at various current densities.

Samples	Specific Capacitance (F g^−1^) at Current Densities (A g^−1^)				
	1	2	3	4	5
N_1_C_2_S_9_-160-15	935	865	821	785	749
N_1.5_C_1.5_S_9_-160-15	983	907	847	794	753
N_2_C_1_S_9_-160-15	1296	1230	1191	1162	1139
N_2.25_C_0.75_S_9_-160-15	1080	1017	957	910	874
N_2_C_1_S_6_-160-15	949	856	805	761	723
N_2_C_1_S_12_-160-15	996	919	857	806	768
N_2_C_1_S_9_-140-15	1103	1020	952	892	849
N_2_C_1_S_9_-150-15	1150	1074	1018	964	922
N_2_C_1_S_9_-170-15	1045	976	913	862	823
N_2_C_1_S_9_-160-9	1082	1020	978	931	896
N_2_C_1_S_9_-160-12	1195	1127	1081	1029	990
N_2_C_1_S_9_-160-18	1055	1004	958	911	872

**Table 3:** Fitted parameters for the Nyquist data obtained from the equivalent circuit model for nickel–cobalt sulfide electrodes.

Samples	R_1_ (Ω)	Q_2_ [F.s^(a_2_ − 1)^]	a_2_	R_2_ (Ω cm^2^)	s_2_ (Ω s^−1/2^ cm^2^)	C_3_ (F)
N_1_C_2_S_9_-160-15	1.095	8.89 × 10^−3^	0.585	0.398	0.314	1.625
N_1.5_C_1.5_S_9_-160-15	1.123	5.85 × 10^−3^	0.635	0.453	0.605	2.104
N_2_C_1_S_9_-160-15	1.231	1.79 × 10^−3^	0.773	0.237	0.591	1.912
N_2.25_C_0.75_S_9_-160-15	1.043	4.85 × 10^−3^	0.552	0.779	0.396	1.203
N_2_C_1_S_9_-140-15	1.077	3.27 × 10^−3^	0.628	0.481	0.350	1.546
N_2_C_1_S_9_-150-15	1.091	4.66 × 10^−3^	0.605	0.526	0.272	1.833
N_2_C_1_S_9_-170-15	1.144	7.22 × 10^−3^	0.575	0.403	0.381	1.320
N_2_C_1_S_6_-160-15	1.129	0.82 × 10^−3^	0.721	0.892	0.380	0.763
N_2_C_1_S_12_-160-15	1.109	1.46 × 10^−3^	0.685	0.864	0.311	0.768
N_2_C_1_S_9_-160-9	1.035	6.50 × 10^−3^	0.609	0.312	0.368	1.254
N_2_C_1_S_9_-160-12	1.148	1.20 × 10^−3^	0.705	0.583	0.684	1.245
N_2_C_1_S_9_-160-18	1.208	3.11 × 10^−3^	0.635	0.415	0.479	1.190

**Table 4:** Comparison of the specific capacitance based on different NiCoS electrodes.

No.	Materials	Substrate	Synthesis Conditions	Electrolyte	Specific Capacitance	Ref.
1	sulfur-deficient NiCo_2_S_4_	Ni foam Direct	Hydrothermal 110 °C, 13 h; Annealed 400 °C, 1 h (Ar atmosphere)	mass ~2.6 mg cm^−2^ 0.0~0.4 V vs. Ag/AgCl 2 M KOH	971 F g^−1^ at 2 A g^−1^	[32]
2	urchin-like NiCo_2_S_4_	Ni foam mixing	Hydrothermal 160 °C, 6 h	mass 2–3 mg 0.0~0.6 V vs. Hg/HgO 6 M KOH	1149 F g^−1^ at 1 A g^−1^	[14]
3	NiCo_2_S_4_ nanocolliods	Nickel foam mixing	Solvothermal 180 °C, 6 h; Sulfurization 120 °C, 8 h	0.0–0.6 V vs. SCE 2 M KOH	935 F g^−1^ at 3 A g^−1^	[33]
4	onion-like NiCo_2_S_4_	Nickel foam mixing	Solvothermal 160 °C, 4 h; thermal treatment 500 °C, 10 min; anion exchange 90 °C, 24 h; cation exchange 160 °C, 4 h	0.0~0.55 V vs. SCE 6 M KOH	1016 F g^−1^ at 2 A g^−1^	[34]
5	r-NiCo_2_S_4_	Nickel foam mixing	Solvothermal 160°C, 4 h; Sulfurization 160 °C, 6 h; reduction, RT, 2 h	area 1 × 2 cm^2^ 0.0~0.55 V vs. Hg/HgO 3 M KOH	763.5C g^−1^ at 1 A g^−1^	[19]
6	NiCo_2_S_4_ ball-in-ball hollow spheres	Nickel foam mixing	solvothermal 180 °C, 6 h; sulfidation 160 °C, 0.5 h; annealed 300 °C, 0.5h (N_2_ atmosphere)	mass 5 mg cm^−2^ −0.1~0.55 V vs. SCE 6 M KOH	1036 F g^−1^ at 1 A g^−1^	[35]
7	NiCoS-12	Carbon cloth Direct	Hydrothermal 120 °C, 14 h; sulfidation 150 °C, 6 h; plasma-treated	area 1 × 1 cm^2^ mass 0.8 mg cm^−2^ −0.1~0.5 V vs. Ag/AgCl 1 M KOH	673.15 F g^−1^ at 1 A g^−1^	[36]
8	hollow nanoneedle NiCo_2_S_4_	Commercial carbon fiber papers Direct	Hydrothermal 90 °C, 5 h; then 140 °C, 5 h	area 1 × 1 cm^2^ 0.1~0.65 V vs. Ag/AgCl 2 M KOH	1154 F g^−1^ at 1 A g^−1^	[37]
9	NCSW-200	Nickel foam mixing	Hydrothermal 200 °C, one day	area 1 × 1 cm^2^ −0.4~0.4 V 6 M KOH	369 F g^−1^ at 0.5 A g^−1^.	[38]
10	NCS/NS-G	carbon fiber paper	Hydrothermal 140 °C, 14 h; hydrothermal 140 °C, 24 h	area 1 × 1 cm^2^ −0.1~0.45 V vs. Ag/AgCl 6 M KOH	630.6 F g^−1^ at 1 A g^−1^	[39]
11	urchin-like Co-doped NiS_2_/C nanorod array	Carbon cloth	Solvothermal 160 °C, 15 h	area 1 × 1 cm^2^ mass 2 mg cm^−2^ −0.09~0.51 V vs. Hg/HgO 3 M KOH	1296 F g^−1^ at 1 A g^−1^	This study

## Data Availability

The original contributions presented in this study are included in the article/supplementary material. Further inquiries can be directed to the corresponding author.

## Supplementary Materials

The following supporting information can be downloaded at https://www.mdpi.com/article/10.3390/ma19122651/s1. Figure S1: SEM images of nickel–cobalt sulfide samples: (a) N_1.5_C_1.5_S_9_-160-15, (b) N_2_C_1_S_9_-150-15, (c) N_2_C_1_S_9_-160-12; Figure S2: CV curves at various scan rates of nickel–cobalt sulfide electrodes; Figure S3: Plots of i vs. v of Co-NiS_2_ electrode synthesized under different Ni:Co ratios; Figure S4: GCD curves at various current densities of nickel–cobalt sulfide electrodes; Figure S5: Nyquist plot of nickel–cobalt sulfide electrodes synthesized under different solvothermal conditions: (a) S:(Ni + Co) ratio, (b) temperature, and (c) time (inset shows the enlarged view of the Nyquist plot in the high frequency region). Table S1: Lattice parameter calculated from the (200) diffraction peak and corresponding uncertainties for the Ni-Co sulfide samples.
