# Supplementary material for: Synergistic Regulation of Composition and Growth Kinetics in Cobalt-Doped Nickel Sulfides for High-Performance Pseudocapacitors
Source: Materials (Basel). 2026 Jun 19;19(12):2651. doi: 10.3390/ma19122651 (PMC13304385; doi:10.3390/ma19122651)
Supplement: Supplementary file 1 [file materials-19-02651-s001.zip › Figure S1.pdf]

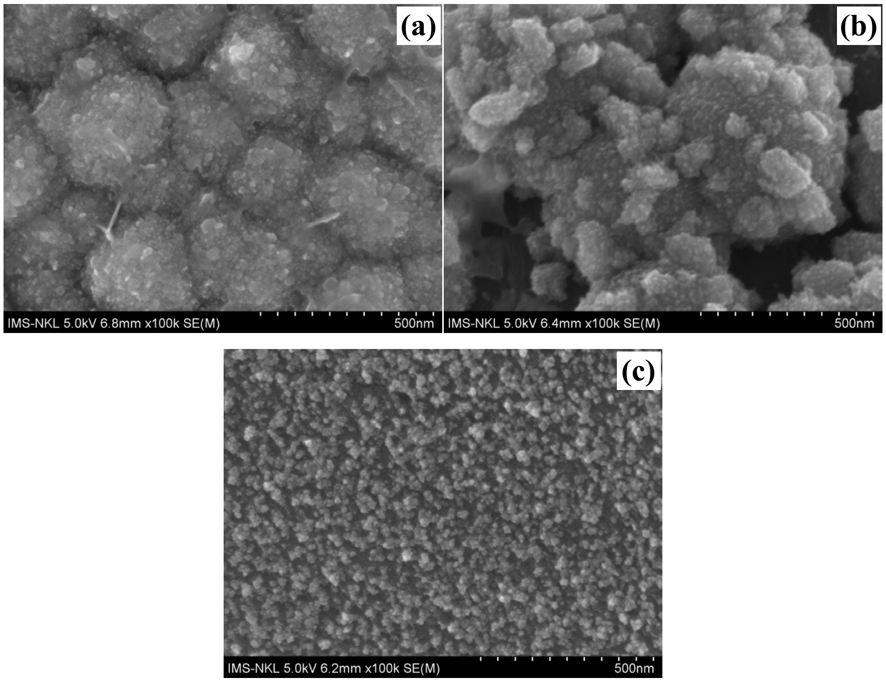

Figure S1. SEM images of nickel cobalt sulfide samples:

(a) $N_{1.5}C_{1.5}S_9$-160-15, (b) $N_2C_1S_9$-150-15, and (c) $N_2C_1S_9$-160-12.
