# Supplementary material for: Synergistic Regulation of Composition and Growth Kinetics in Cobalt-Doped Nickel Sulfides for High-Performance Pseudocapacitors
Source: Materials (Basel). 2026 Jun 19;19(12):2651. doi: 10.3390/ma19122651 (PMC13304385; doi:10.3390/ma19122651)
Supplement: Supplementary file 1 [file materials-19-02651-s001.zip › Figure S5.pdf]

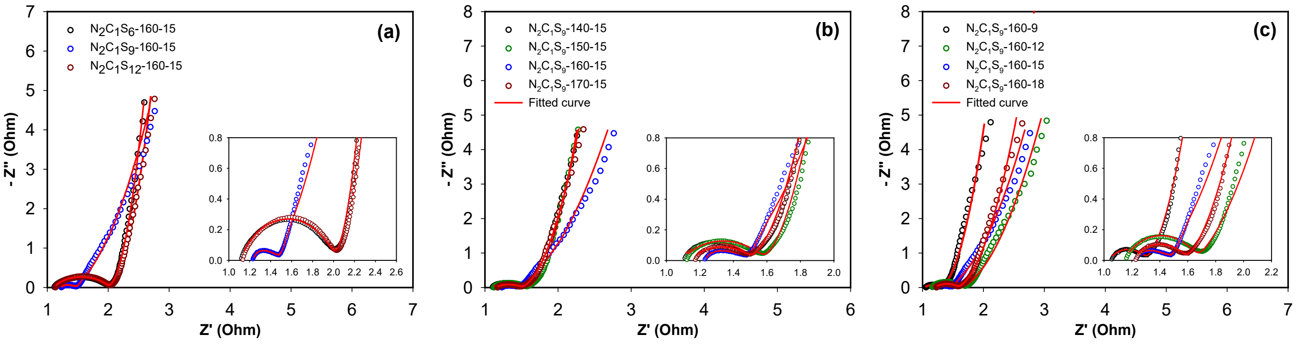

Figure S5. Nyquist plot of nickel cobalt sulfide electrodes synthesized under different solvothermal conditions: (a) S:(Ni+Co) ratio, (b) temperature, and (c) time (Inset shows the enlarged view of the Nyquist plot in the high frequency region).
