# Supplementary material for: Synergistic Regulation of Composition and Growth Kinetics in Cobalt-Doped Nickel Sulfides for High-Performance Pseudocapacitors
Source: Materials (Basel). 2026 Jun 19;19(12):2651. doi: 10.3390/ma19122651 (PMC13304385; doi:10.3390/ma19122651)
Supplement: Supplementary file 1 [file materials-19-02651-s001.zip › Table S1.pdf]

Table S1. Lattice parameter calculated from the (200) diffraction peak and corresponding uncertainties for the Ni-Co sulfide samples.

Sample	a (Å)	$\Delta(2\theta)$ (°)	Δa (Å)	Lattice parameter (Å)
$N_1C_2S_9$-160-15	5.5804	0.00613	0.0010	5.5804±0.0010
$N_{1.5}C_{1.5}S_9$-160-15	5.6068	0.00414	0.0007	5.6068±0.0007
$N_2C_1S_9$-160-15	5.6277	0.00300	0.0005	5.6277±0.0005
$N_{2.25}C_{0.75}S_9$-160-15	5.6407	0.00402	0.0007	5.6407±0.0007
