# Supplementary figures and images for: Synergistic Regulation of Composition and Growth Kinetics in Cobalt-Doped Nickel Sulfides for High-Performance Pseudocapacitors
Source: Materials (Basel). 2026 Jun 19;19(12):2651. doi: 10.3390/ma19122651 (PMC13304385; doi:10.3390/ma19122651)

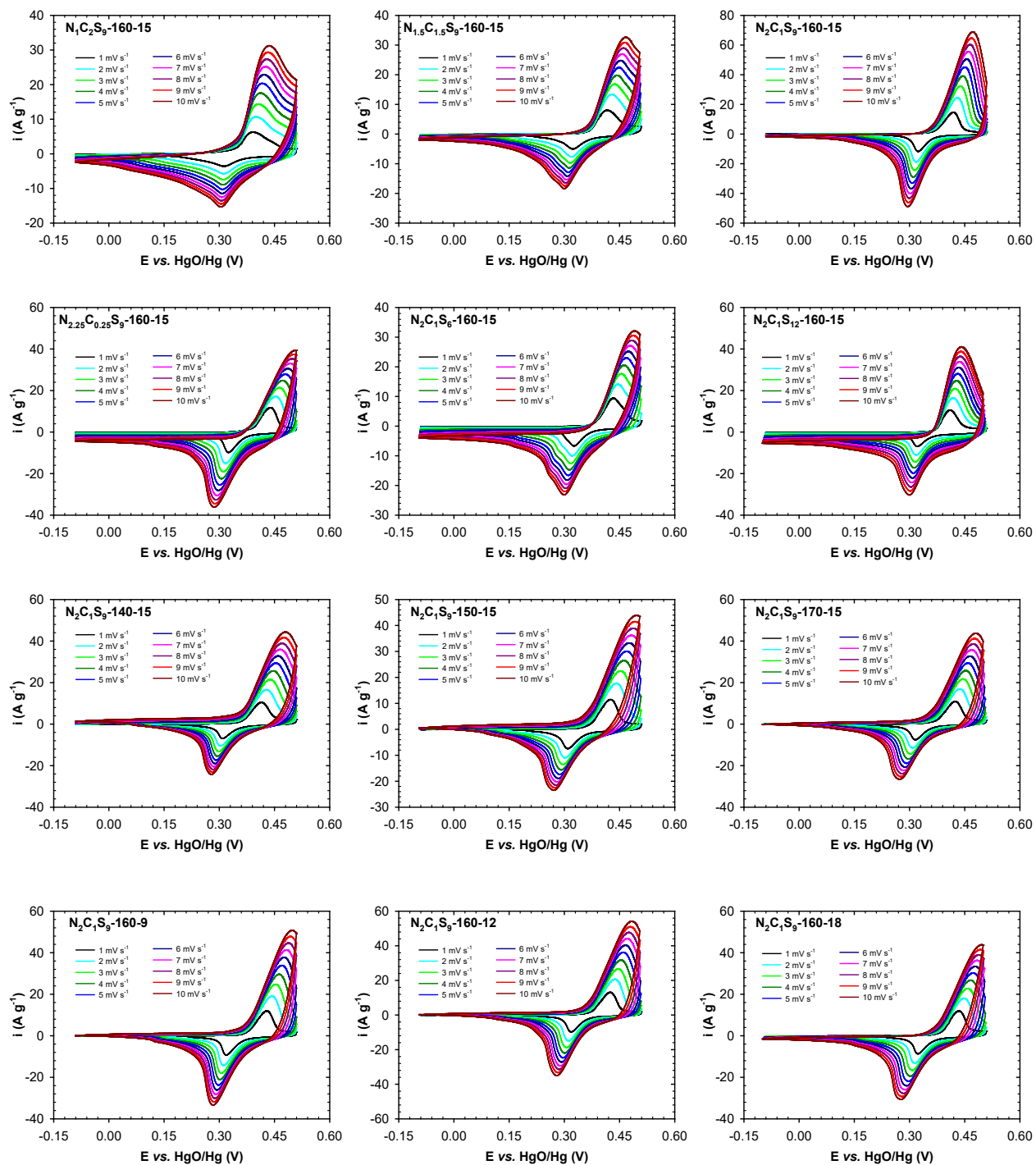

Figure S2. CV curves at various scan rates of nickel cobalt sulfide electrodes.

Supplement: Supplementary file 1 [file materials-19-02651-s001.zip › Figure S2.pdf]

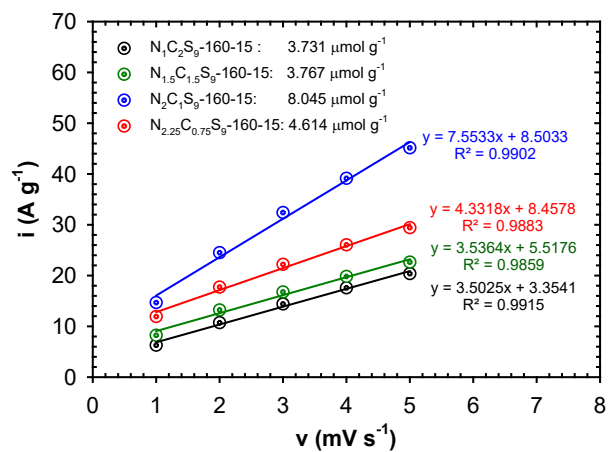

**Figure S3.** Plots of  $i$  vs.  $v$  of Co-NiS<sub>2</sub> electrode synthesized under different Ni:Co ratios.

Supplement: Supplementary file 1 [file materials-19-02651-s001.zip › Figure S3.pdf]

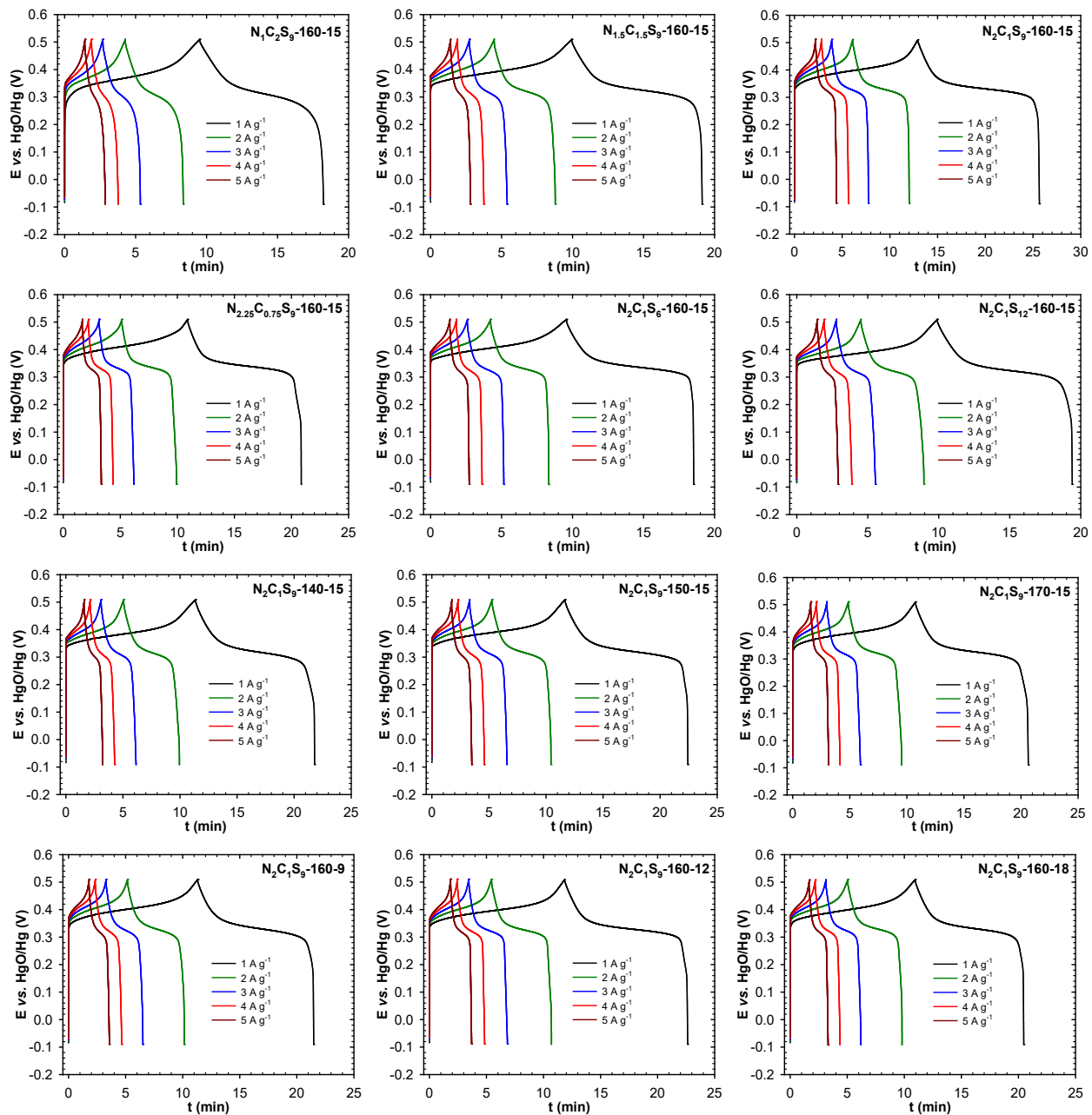

Figure S4. GCD curves at various current densities of nickel cobalt sulfide electrodes.

Supplement: Supplementary file 1 [file materials-19-02651-s001.zip › Figure S4.pdf]
